# A multicentre survey investigating the knowledge, behaviour, and attitudes of surgical healthcare professionals to frailty assessment in emergency surgery: DEFINE(surgery)

**DOI:** 10.1007/s41999-024-00962-7

**Published:** 2024-04-18

**Authors:** P. Braude, F. Parry, K. Warren, E. Mitchell, K. McCarthy, R. G. Khadaroo, B. Carter, CLARITY (collaborative ageing research) group, Nia Humphry, Sara Long, Heeam Nassa, Arturo Vilches-Moraga, Nahida Bashir, Ashly Thomas, Frances Rickard, Mike Sellick, Paolo Buscemi, Hwei Jene Ng, Terry Quinn, Katrina Knight, Eilidh Bruce, Phyo Kyaw Myint

**Affiliations:** 1 CLARITY (Collaborative Ageing Research) group, North Bristol NHS Trust, Bristol, UK; 2 Department of Urology, North Bristol NHS Trust, Bristol, UK; 3 Colorectal Cancer and Surgery, North Bristol NHS Trust, Bristol, UK; 4 Department of Surgery and Critical Care Medicine, University of Alberta, Edmonton, Alberta Canada; 5 Department of Biostatistics and Health Informatics, Institute of Psychiatry, Psychology and Neuroscience, King’s College London, De Crespigny Park, London, UK; 6 Centre for Health and Clinical Research, University of the West of England, Bristol, UK

**Keywords:** Frailty, Perioperative medicine, Surgery, Attitudes, National survey

## Abstract

**Purpose:**

Screening for frailty in people admitted with emergency surgical pathology can initiate timely referrals to enhanced perioperative services such as intensive care and geriatric medicine. However, there has been little research exploring surgical healthcare professionals' opinions to frailty assessment, or accuracy in identification. This study aimed to assess the knowledge, behaviour, and attitudes of healthcare professionals to frailty assessment in emergency surgical admissions.

**Methods:**

We designed a cross-sectional multicentre study developed by a multiprofessional team of surgeons, geriatricians, and supported by patients. A semi-structured survey examined attitudes and behaviours. Knowledge was assessed by comparing respondents’ accuracy in scoring twenty-two surgical case vignettes using the Clinical Frailty Scale.

**Results:**

Eleven hospitals across England, Wales, and Scotland participated. Two hundred and eleven clinicians responded—20.4% junior doctors, 43.6% middle grade doctors, 24.2% senior doctors, 11.4% nurses and physician associates. Respondents strongly supported perioperative frailty assessment. Most were already assessing for frailty, although frequently not using a standardised tool. There was a strong call for more frailty education. Participants scored 2175 vignettes with 55.4% accurately meeting the gold standard; accuracy improved to 87.3% when categorised into “not frail/mildly frail/severely frail” and 94% when dichotomised to “not frail/frail”.

**Conclusion:**

Frailty assessment is well supported by healthcare professionals working in surgery. However, standardised tools are not routinely being used, and only half of respondents could accurately identify frailty. Better education around frailty assessment is needed for healthcare professionals working in surgery to improve perioperative pathway for people living with frailty.

**Supplementary Information:**

The online version contains supplementary material available at 10.1007/s41999-024-00962-7.

## Key summary points

**Aims:**

This study aimed to assess the knowledge, behaviour, and attitudes of healthcare professionals to frailty assessment in emergency surgical admissions.

**Findings:**

Frailty assessment is well supported by healthcare professionals working in surgery. However, in clinical practice, standardised tools are not routinely being used and only half of respondents were able to accurately identify frailty from clinical vignettes.

**Message:**

Better education around frailty assessment is needed for healthcare professionals working in surgery to improve perioperative pathway for people living with frailty.

**Supplementary Information:**

The online version contains supplementary material available at 10.1007/s41999-024-00962-7.

## Introduction

How do surgeons view frailty, and can they assess for it? Older adults living with frailty represent an increasingly large proportion of surgical admissions [1]. A systematic review identified between 1 in 10 and 1 in 3 of general surgical patients were living with frailty, with the proportion varying based on the included population and tool used [2]. In any setting, frailty is associated with worse post admission outcomes including more complications, longer hospital stay, and higher mortality [3]. Frailty screening may allow early referral to enhanced services such as intensive care and multidisciplinary teams working with geriatricians. This has led to national guidance and NHS incentive-based schemes linking funding with frailty assessment including in emergency laparotomy and major trauma [4–6]. Assessment for frailty by a geriatrician amongst patients being admitted with major trauma has been shown to reduce inpatient mortality [7]. The likely mechanism being the identification of frailty activating a comprehensive geriatric assessment and management [8, 9].

However, it is not known if clinicians working within surgical disciplines are supportive of frailty assessment, find it useful in perioperative planning, or are accurately screening for its presence. Investigating this is key to frailty screening becoming embedded into routine surgical practice, without the perpetual need for financial drivers, and ultimately ensuring the right patients receive the right frailty attuned care early in admission. In Canada, a survey of 49 healthcare workers in a single surgical unit showed that professionals lacked confidence and knowledge in using frailty assessment tools and found these to be barriers in their use [10]. In the UK, a survey at a surgical conference demonstrated doctors felt inadequately trained in perioperative medicine to manage complex, older surgical patients [11]. The aim of this study is to explore the knowledge, behaviours, and attitudes of healthcare professionals to frailty assessment of older people when admitted with an emergency surgical issue.

## Methods

The study has been written in concordance with the CHERRIES reporting statement [12].

The aims of this study were to:

1. Evaluate the ability of healthcare professionals involved in the care of patients admitted under surgical services to accurately assess frailty in comparison to a gold standard of a geriatrician-assessed frailty score.
2. Explore healthcare professionals’ self-reported practice of frailty assessment of older adults admitted to a surgical service, including: (i) perceived importance of assessing frailty, (ii) perceived usefulness of specific frailty scale scores, (iii) differences in perspectives between specialties and seniority, (iv) educational needs of different healthcare professional groups.

### Design

A multicentre cross-sectional study called Decisions Encompassing Frailty In Novel Environments—DEFINE(surgery) was designed to target healthcare professionals involved in assessing emergency surgical admissions. It was designed in two parts: (1) Clinical vignettes to be scored to assess the ability of healthcare professionals to accurately assess for frailty and (2) a semi-structured survey to explore the attitudes and behaviours to frailty assessment in the perioperative setting.

Participants were eligible to take part if they had worked in a surgical specialty for at least one month, were involved in acute assessment of patients admitted under a surgical department, and were a doctor, nurse, or physician associate of any level of seniority.

### Ethical approval and consent process

The study was funded by the British Geriatric Society and was sponsored by North Bristol NHS Trust (R&I ID 5002). It was approved by the Human Research Authority (IRAS number 309808). Ethical approval was not required under paragraph 2.3.14 of Governance arrangements for Research Ethics Committees as only healthcare staff were being recruited through their professional role.

A participant information sheet and consent form were included as part of the first page of the online survey. To keep the survey short, a lean dataset was collected including minimal personal data. Data were stored on secure servers at North Bristol NHS Trust.

### Development and pre-testing

Twenty-two short clinical case vignettes were created to assess the ability of healthcare professionals to accurately assess frailty using the Clinical Frailty Scale (CFS). Three geriatricians created clinically relevant case vignettes through iterative discussion. The vignettes covered the full range of levels of frailty within the CFS from 1 to 9. The number of vignettes for each level of frailty were proportionally distributed as reported in the general surgical literature [2]. The vignettes were validated independently by three pairs of consultant geriatricians, none of whom were involved in the study design. Two surgeons reviewed the vignettes for clinical accuracy and performed a first pilot. Any disagreements were discussed by the study team and adjustments made. The Acute Frailty Networks’ CFS App was used to recheck all scores. The semi-structured survey was adapted for a UK cohort from a similar Canadian study [15].

The study group conducted a second pilot of the full survey with doctors working in the North Bristol NHS Trust urology department. All 22 vignettes were presented; the proportion completed, and time taken to complete were recorded. Comments from the pilot participants were mainly around the layout and time to complete the vignettes. For the final survey, the burden of completion was reduced through reformatting and reducing the number of vignettes presented to any single participant. To reduce the time taken to complete while still maintaining a full range of frailty levels scored, twelve vignettes were selected at random for each site from a bank of 22, which were presented in a random order to each participant.

### Patient involvement

Three patients from the CLARITY (Collaborative Ageing Research) patient feedback group were invited to contribute to the study. They were interviewed focusing on the study and frailty in the perioperative period. Feedback from the responses was used to design the protocol and the study materials.

### Recruitment process

The study group identified 11 hospitals from across the UK. Sites were invited via a UK wide existing research network called OPSOC (Older Persons Surgical Outcomes Collaborative) and advertised through the NIHR Clinical Research Network. This was a closed survey with participants invited by each site’s surgical lead or leads. A template email invitation for participants and reminders were distributed by the central team. Sites advertised the presence of the survey within departments. The closed nature meant the survey could be targeted at the study population only and a final sample completion rate could be calculated. Invitations were sent by email to potential participants from the local surgical lead(s) with a site-specific weblink. The study was promoted on social media but without any site-specific links.

Eleven hospitals from three countries of the UK participated, including: In Wales-University Hospital of Wales, Cardiff and Vale University Health Board; The Grange, Aneurin Bevan University Health Board. In England-Southmead Hospital, North Bristol NHS Trust; Salford Royal Infirmary, Northern Care Alliance NHS Foundation Trust; Royal United Hospitals NHS, Bath; Musgrove Park, Somerset Foundation Trust; Bristol Royal Infirmary, University Hospitals Bristol and Weston NHS Foundation Trust. In Scotland—Royal Alexandra Hospital, Royal Inverclyde Hospital, and Glasgow Royal Infirmary, NHS Greater Glasgow and Clyde; Aberdeen Royal Infirmary, NHS Grampian.

### Survey administration

Data were collected using an online survey (SurveyMonkey from Momentive). Responses were recorded through a combination of radio buttons, free text boxes, and pull-down menus. Completion of the survey was voluntary with no incentives provided. The survey was live for a minimum of two weeks at each site, with all sites closed by August 2022. The behaviour and attitudes survey were presented first, followed by the vignettes. Each vignette was presented with a pictorial version of the CFS v2.0 [13]. The survey contained 37 questions over 15 pages. The only mandatory sections to advance in the survey were the fields to confirm eligibility and consent on the first page. No completeness checks occurred before submission. Participants were able to review and change their answers using forward and back buttons, with no summary was presented for answer review before submission.

### Response rates

In total, 994 participants were invited to participate. One site had missing data for the number of participants invited. The recruitment rate was 20.5% (proportion of recruited from all participants invited). Median site recruitment rate was 25.1% (IQR 14.0–36.2%). The overall mean survey completion rate, from consenting page to final question, was 83.6%. The median completion rate for each site was 86% (IQR 77.5–91.5%). The median time taken to complete each survey was 9 min 12 s (IQR 8 min 14 s–9 min 53 s). Duplicate entries were filtered out prior to data extraction by the online survey programme using cookies. No other unique identifiers were collected.

## Analysis

Records were excluded if either submitted before, or after the site-specific survey was opened, or if all data were missing after the consenting page. Surveys with incomplete fields still had available data extracted.

For the vignettes, each hospital lead was sent a randomised set of 12 out of 22 vignettes. The respondents’ scores of the 22 vignettes were compared against the geriatrician validated scores as the gold standard. Frailty scores were examined with three methods—at each level of the CFS 1–9 and categorised into three groups: not frail (CFS 1–4), mild-to-moderate frailty (CFS 5–6), severe frailty (CFS 7–8), and (CFS 9) a terminal illness but not otherwise severely frail, and categorised into two groups: not frail (CFS 1–4), and living with frailty (CFS 5–9).

For qualitative analysis of the semi-structured survey, professionals were categorised into four groups: junior doctors (Foundation Year 1 or 2, and Senior House Officers—approximately 1–3 years postgraduate), middle grade doctors (Core Trainees 1–3, Specialist Trainees 3–8, and equivalent—approximately 3–8 years postgraduate), senior doctors (consultant, and equivalent), and nurses and physician associates. For descriptive analysis, we calculated mean values and standard deviations.

### Results of knowledge and behaviours survey

Across all 11 sites between 11th May and 27th July 2022 a total of 211 healthcare professionals consented to participate in the survey. This included 43 (20.4%) junior doctors, 92 (43.6%) middle grade doctors, 51 (24.2%) senior doctors, and 24 (11.4%) nurses and physician associates, with one respondent’s role missing (Table 1). There were 16 respondents’ data excluded having not completed any of the vignettes or survey. One hundred and ninety-five respondents were included in the final analysis. The majority of the clinicians were from general surgery (*n* = 158, 74.9%), with other specialties evenly split between trauma and orthopaedics, urology, and vascular surgery. Most clinicians had been working for over two years in surgical specialities (*n* = 146, 68.5%).

**Table 1:** Characteristics of the included healthcare workers

	A frailty assessment should be done for all surgical patients				
	Strongly agree /agree (*n* = 147)	Neither agree or disagree (*n* = 12)	Strongly disagree/disagree (*n* = 36)	Missing (*n* = 16)	Total (*n* = 211)
*Time in surgical specialty*					
0–4 months	24 (85.7)	2 (7.1)	2 (7.1)	6	34 (16.0)
5–12 month	13 (94.1)		1 (5.9)	2	19 (8.9)
1–2 years	7 (58.3)		5 (41.7)		12 (5.6)
> 2 years	100 (68.5)	10 (6.9)	28 (19.2)	8	146 (68.5)
*Grade*					
Junior doctor	30 (85.7)	2 (5.7)	3 (8.6)	8	43 (20.4)
Middle grade doctor	62 (69.7)	7 (7.9)	20 (22.5)	3	92 (43.6)
Senior doctor	35 (72.9)	3 (6.3)	10 (20.8)	3	51 (24.2)
Nurse/physician associate	19 (86.4)		3 (13.6)	2	24 (11.4)
Missing	1				1
*Specialty*					
General surgery	112 (70.9)	9 (5.7)	25 (15.8)	12	158 (74.9)
Urology	12 (70.6)	1 (6.9)	3 (17.7)	1	17 (8.1)
Vascular	11 (68.8)		3 (18.8)	2	16 (7.6)
Trauma and orthopaedics	8 (61.5)	2 (15.4)	3 (23.1)		13 (6.2)
Other	4 (66.7)		2	1	7

Three quarters of participants (*n* = 147, 75.4%) agreed that a frailty assessment should be done for all older surgical patients (Table 1), with the vast majority (88.7%) expressing that it was part of their role to assess patients for frailty (Supplementary Table 1). Almost all (99.5%), with only one exception, agreed that a patient’s level of frailty should play a role in the perioperative care plan. However, under half agreed (44.1%) that a patient’s level of frailty always played a role in their own planning. A third of participants always used a tool when assessing frailty (36.9%), but this was highest for nurses and physician associates (54.5%). Around half of the sample selected “no tool, just make a judgement” to assess for frailty. Overall, two-thirds of healthcare professionals were confident in their ability to assess patients for frailty, with confidence highest for nurses and physician associates (77.3%), and increasing with seniority of doctor (senior doctors 62.5% vs. junior doctors 48.6%).

In questions focussed on the Clinical Frailty Scale (CFS) only, most participants had experience of using the CFS to assess frailty (*n* = 118, 55.4%). Half of respondents (48.7%) agreed it was useful in the perioperative care they provided in hospital, and half agreed it was useful to the overall perioperative pathway (51.8%). About half of the sample (55.9%) expressed that they would like to use or continue to use the CFS to score frailty (Supplementary Table 2). Over a third (39.5%) agreed they would benefit from further training on how to use the CFS, with junior staff more frequently wanting more training (51.4%).

### Results of the vignette frailty scoring

Across the 195 respondents, 92.7% (2175/2342) vignettes were scored. Just over half of the assessments matched the gold standard (1205/2175, 55.4%, Table 2). Across the 22 vignettes, the median percentage that matched correctly to the gold standard was 54% (IQR, 48–63%).

**Table 2:**
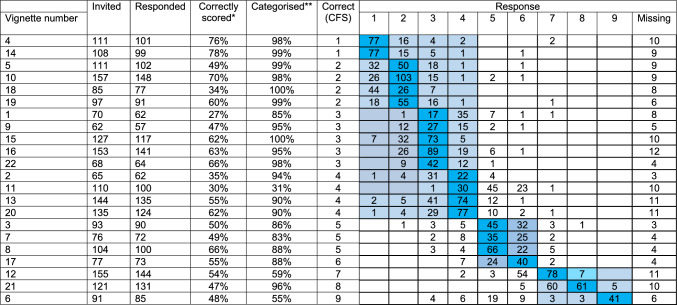
The accuracy of participants frailty scores of case vignettes against a gold-standard geriatrician assessed score

Median (IQR), *median = 54% (48–63%); **median = 94% (87–98%)

When assessing the frailty score grouped into not frail, mildly or moderate frail, and severely frail (including CFS 9) 87.3% (1900/2175) matched the categorical group. Across the 22 vignettes the median percentage that matched the gold standard when frailty scores were categorised into not frail, and frail was 94% (87–98%).

Only three vignettes were typically assessed not matching the categorised score from the gold standard (Vignettes 6, 11, and 12). Vignette 6 (CFS 9) was matched by 55%, vignette 11 (CFS 4) by 31%, and vignette 12 (CFS 7) by 59%.

## Discussion

This study has robustly examined the current state of the surgical healthcare professionals’ knowledge, behaviour, and attitudes towards frailty assessment across the UK. We have demonstrated that frailty assessment is strongly viewed as a part of surgeons’ clinical responsibility, and frequently used in perioperative care planning. Most participants have employed frailty tools in clinical practice, with the CFS being most commonly used, but many respondents are assessing for frailty without a standardised tool. Respondents identified a need for more training in frailty assessment, including using the CFS, despite it being the most widely used tool already. When assessing for frailty using the CFS with clinical vignettes, only about half were accurately scored. Accuracy greatly improved by di- and trichotomising the frailty severity levels within the CFS.

These findings have significant implications for acute surgical pathways of care. We have shown that frailty assessment is well supported by surgical healthcare professionals. This wide support should help frailty screening become integral to emergency surgical admissions. Awareness of frailty assessment has been driven by promotion in national guidelines: in 2020 NICE guidelines included the CFS as part of algorithmic escalation decision-making to intensive care for people with COVID-19, [14] more recently frailty screening has been promoted by multiple surgical and national bodies linking completion of frailty scores with financially incentivised targets.

Despite there being a high level of support for frailty assessment, many surgeons indicated that they are not always using a standardised frailty tool. Making a judgment, or “eyeball testing” frailty, most frequently was selected by doctors compared to nurses and physician associates. This method of frailty assessment has been shown to be unreliable in comparison to standardised tools and should not be an acceptable substitute [15, 16].

When testing the ability of respondents to apply the most used tool, the CFS, these data demonstrated frailty status was only correctly identified half of the time. In practice, this may lead to many patients being incorrectly labelled as frail or not frail and experience a varying clinical response. Accuracy of identifying a person living with frailty increases when using categories of the CFS both through dichotomising “not frail / frail”, or trichotomising “not frail / mild-to-moderately frail/severely frail”, rather than relying on an exact frailty score to activate a pathway.

Respondents strongly indicated that they would like more education around frailty assessment. This finding is not reflected in the current surgical training curricula, such as from the Royal College of Surgeons, which does not contain any references to frailty [17]. As our population ages, and the proportion of older people in emergency surgical admissions increases, frailty screening will become more relevant in clinical decision making. Additional education may improve uptake of standardised frailty tools, as well as their accurate application.

The strengths of this study are the piloting of the survey with a multiprofessional group and patient input, as well as validation through multiple rounds of iterative improvement. We included a large number of hospitals across three nations of the UK. In addition, a large number of participants completed the survey and vignettes with a good spread of recruitment across sites, professions, and levels of seniority. The main limitations of this study included possible recruitment bias with only participants knowledgeable or interested in frailty taking time to complete the survey. Many of the included sites had some form of geriatric perioperative medicine service already, with the possible effect of having disseminated and embedded frailty assessment in clinical practice more than a site with no geriatrician input. Despite this some participants did not provide consistent responses to the frailty scoring indicating possible misclassification and survey response fatigue. In the analysis the categorisation of CFS 9 remains problematic given it is akin to CFS 1–6 and not 7–8, given the definition is “not living with severe frailty”. We chose to include CFS in the frail group as people that may receive a CFS 9 may still benefit from a holistic assessment by a geriatrician. The generalisability of the findings maybe further limited by the majority of staff working in general surgery and few from other disciplines.

Future research should look at whether these survey findings reflect the use of frailty assessment in clinical practice, and how clinical decision making is altered when frailty is identified. Patients and carers views should also be explored to deliver a coordinated and valued response to the identification of frailty.

## Conclusion

This study has demonstrated that frailty assessment is well supported in surgical healthcare professionals’ practice. However, standardised frailty tools are not routinely being used, with only half of clinicians accurately identifying frailty. Clinicians want more education around frailty assessment, which may improve the reliability in identifying frailty. Using categorisation of frailty scores can immediately improve the accuracy for triggering specialised frailty attuned pathways of care.

## Supplementary Information

Below is the link to the electronic supplementary material.

Supplementary file1 (DOCX 17 kb)
