# Supplementary material for: A multicentre survey investigating the knowledge, behaviour, and attitudes of surgical healthcare professionals to frailty assessment in emergency surgery: DEFINE(surgery)
Source: Eur Geriatr Med. 2024 Apr 18;15(4):1047–53. doi: 10.1007/s41999-024-00962-7 (PMC11377612; doi:10.1007/s41999-024-00962-7)
Supplement: Supplementary file 1 — Supplementary file1 (DOCX 17 kb) [file 41999_2024_962_MOESM1_ESM.docx]

Supplementary Table 1 - Views and opinions of the participants from the included sample

|  | Foundation Doctor  (n=35) | Middle Grade  (n=89) | Consultant  (n=48) | Nurse/Physician Associate  (n=22) | Total  (n=194*) |
| --- | --- | --- | --- | --- | --- |
| It is part of my professional role/responsibility to assess patients for frailty | | | |  |  |
| Agree | 29 (82.9) | 78 (87.6) | 43 (89.6) | 22 (100) | 172 (88.7) |
| Neither agree or disagree | 4 (11.4) | 6 (6.7) | 3 (6.3) |  | 13 (6.7) |
| Disagree | 2 (5.7) | 5 (5.6) | 2 (4.2) |  | 9 (4.6) |
|  |  |  |  |  |  |
| I always use a frailty assessment tool when assessing patients for frailty | | | |  |  |
| Agree | 11 (31.4) | 28 (31.5) | 20 (41.7) | 12 (54.5) | 71 (36.6) |
| Neither agree or disagree | 5 (14.3) | 11 (12.4) | 6 (12.5) | 5 (22.7) | 27 (13.9) |
| Disagree | 19 (54.3) | 49 (55.1) | 22 (45.8) | 4 (18.2) | 94 (48.5) |
| Missing |  | 1 (1.1) |  | 1 | 2 (1.0) |
|  |  |  |  |  |  |
| I am confident in my ability to assess patients for frailty | | | |  |  |
| Agree | 17 (48.6) | 53 (59.6) | 30 (62.5) | 17 (77.3) | 117 (60.3) |
| Neither agree or disagree | 7 (20.0) | 23 (25.8) | 10 (20.8) | 4 (18.2) | 44 (22.7) |
| Disagree | 11 (31.4) | 13 (14.6) | 8 (16.7) | 1 (4.6) | 33 (17.0) |
|  |  |  |  |  |  |
| A patient’s level of frailty should play a role in planning perioperative care in hospital | | | |  |  |
| Strongly Agree | 24 (68.6) | 52 (58.4) | 31 (64.6) | 16 (72.7) | 123 (63.4) |
| Agree | 11 (31.4) | 37 (41.6) | 17 (35.4) | 5 (22.7) | 70 (36.0) |
| Neither agree or disagree |  |  |  | 1 (4.6) | 1 (0.5) |
| Disagree |  |  |  |  |  |
|  |  |  |  |  |  |
| A patient’s level of frailty always plays a role in my planning of their perioperative care in hospital | | | | |  |
| Agree | 16 (45.7) | 42 (47.2) | 21 (43.8) | 6 (27.3) | 85 (43.8) |
| Neither agree or disagree | 11 (31.4) | 6 (6.7) | 3 (6.3) | 5 (22.7) | 25 (12.9) |
| Disagree | 8 (22.9) | 41 (46.1) | 24 (50.0) | 11 (50.0) | 84 (43.3) |
|  |  |  |  |  |  |
| A patient’s level of frailty is an important factor in how I provide their perioperative care in hospital | | | | |  |
| Agree | 23 (65.7) | 52 (58.4) | 23 (47.9) | 7 (31.8) | 105 (54.1) |
| Neither agree or disagree | 7 (20.0) | 5 (5.6) | 4 (8.3) | 5 (22.7) | 21 (10.8) |
| Disagree | 5 (14.3) | 32 (36.0) | 20 (41.7) | 10 (45.5) | 67 (34.5) |
| Missing |  |  | 1 |  |  |
|  |  |  |  |  |  |
| I face barriers to providing in-hospital care for patients who are living with frailty | | | |  |  |
| Agree | 12 (34.3) | 28 (31.5) | 11 (22.9) | 4 (18.2) | 55 (28.4) |
| Neither agree or disagree | 11 (31.4/89) | 28 (31.5) | 13 (27.1) | 10 (45.5) | 62 (32.0) |
| Disagree | 12 (34.3) | 33 (37.1) | 23 (47.9) | 8 (36.4) | 76 (39.2) |

*Note: 1 respondent did not complete this part of the survey

Supplementary Table 2 - Views on using the CFS

|  | Foundation Doctor  (n=35) | Middle Grade  (n=89) | Consultant  (n=48) | Nurse/Physician Associate  (n=22) | Total  (n=194*) |
| --- | --- | --- | --- | --- | --- |
| The CFS Score is useful to the perioperative care that I provide in hospital | | | |  |  |
| Agree | 16 (45.7) | 45 (50.6) | 24 (50.0) | 9 (40.9) | 95 (49.0) |
| Neither agree or disagree | 12 (34.3) | 24 (27.0) | 11 (22.9) | 9 (18.2) | 56 (28.9) |
| Disagree | 7 (20.0) | 19 (21.4) | 13 (27.1) | 4 (18.2) | 43 (22.2) |
|  |  |  |  |  |  |
| The CFS score is useful to the overall perioperative care pathway that is provided in hospital | | | | |  |
| Agree | 14 (40.0) | 54 (60.7) | 23 (47.9) | 9 (40.9) | 101 (52.1) |
| Neither agree or disagree | 9 (25.7) | 21 (23.6) | 11 (22.9) | 8 (36.4) | 49 (25.3) |
| Disagree | 12 (34.3) | 13 (14.6) | 14 (29.2) | 5 (22.7) | 44 (22.7) |
|  |  |  |  |  |  |
| I would like to use, or continue using, the CFS score for adults living with frailty | | | |  |  |
| Agree | 21 (60.0) | 54 (60.7) | 23 (47.9) | 4 (45.5) | 109 (56.0) |
| Neither agree or disagree | 4 (11.4) | 14 (15.7) | 8 (16.7) | 4 (18.2) | 30 (15.5) |
| Disagree | 10 (28.6) | 20 (22.5) | 17 (35.4) | 8 (36.4) | 55 (28.4) |
| Missing |  | 1 |  |  | 1 (0.5) |
|  |  |  |  |  |  |
| I would benefit from further training on how to use the CFS | | | |  |  |
| Agree | 18 (51.4) | 33 (37.1) | 20 (41.7) | 5 (22.7) | 77 (39.7) |
| Neither agree or disagree | 3 (8.6) | 21 (23.6) | 9 (18.8) | 6 (27.3) | 39 (20.1) |
| Disagree | 14 (40.0) | 34 (38.2) | 19 (39.6) | 10 (45.5) | 77 (40.0) |

*Note: 1 respondent did not complete this part of the survey
